# Mobile learning in medicine: an evaluation of attitudes and behaviours of medical students

**DOI:** 10.1186/s12909-018-1264-5

**Published:** 2018-06-27

**Authors:** Thomas J. G. Chase, Adam Julius, Joht Singh Chandan, Emily Powell, Charles S. Hall, Benedict Lyle Phillips, Ryan Burnett, Deborah Gill, Bimbi Fernando

**Affiliations:** 1 University College London, Gower St, London, WC1E 6BT UK; 2 Respiratory Medicine, St Mary’s Hospital, Paddington, London, W2 1NY UK; 3 General Surgery, Queen Elizabeth Hospital Birmingham, Birmingham, B15 2TH UK; 4 Medicine, Chelsea and Westminster Hospital NHS Foundation Trust, London, SW10 9NH UK; 5 The Royal London Hospital, Whitechapel road, London, E1 1BB UK; 6 Guy’s and St Thomas’ Foundation Trust, Department of Nephrology and Transplantation, London, SE1 9RT UK; 7 Royal Infirmary of Edinburgh, Medicine of the Elderly, Old Dalkeith Road, Edinburgh, EH16 4SA UK; 8 Academic Centre for Medical Education, University College London Medical School, London, UK; 9 Royal Free London NHS Foundation Trust, Transplant Surgery, London, NW3 2QG UK

**Keywords:** Electronic learning, Medical students, Learning and study skills, Medical education

## Abstract

**Background:**

Mobile learning (mLearning) devices (such as tablets and smartphones) are increasingly part of the clinical environment but there is a limited and somewhat conflicting literature regarding the impact of such devices in the clinical learning environment. This study aims to: assess the impact of mLearning devices in the clinical learning environment on medical students’ studying habits, attitudes towards mobile device supported learning; and the perceived reaction of clinicians and patients to the use of these devices as part of learning in the clinical setting.

**Methods:**

Over three consecutive academic years, 18 cohorts of medical students (total *n* = 275) on a six-week rotation at a large teaching hospital in London were supplied with mLearning devices (iPad mini) to support their placement-based learning. Feedback on their experiences and perceptions was collected via pre- and post-use questionnaires.

**Results:**

The results suggest mLearning devices have a positive effect on the students’ perceived efficiency of working, while experience of usage not only confirmed pre-existing positive opinions about devices but also disputed some expected limitations associated with mLearning devices in the clinical workplace. Students were more likely to use devices in ‘down-time’ than as part of their clinical learning. As anticipated, both by users and from the literature, universal internet access was a major limitation to device use. The results were inconclusive about the student preference for device provision versus supporting a pre-owned device.

**Conclusion:**

M-learning devices can have a positive impact on the learning experiences medical students during their clinical attachments. The results supported the feasibility of providing mLearning devices to support learning in the clinical environment. However, universal internet is a fundamental limitation to optimal device utilisation.

**Electronic supplementary material:**

The online version of this article (10.1186/s12909-018-1264-5) contains supplementary material, which is available to authorized users.

## Background

Portable electronic devices, including tablet computers and smartphones, are transforming the healthcare environment and are impacting the landscape of medical education [1, 2] with some reports suggesting almost universal ownership of a tablet or smartphone by medical students [3]. These devices can provide learners with easy access to a wide variety of educational resources to support learning in the clinical environment. Mobile devices are used by medical students to facilitate access to a wide range of resources including anatomy, drug information, clinical scoring systems and eBooks (Electronic Books) [4–6] with higher levels of use of devices by students in the clinical years [7]. However, for mLearning to be successfully integrated into medical education strategies and practices, institutions, clinicians and students must understand their potential, impacts and limitations.

Students’ have reported that mLearning initiatives have a positive influence on learning [8–10]. Moreover, students report that mLearning tools have been as effective as traditional teaching in both clinical settings and formal learning environments [10–14]. Students valued the quick and easily accessible information [15–18] afforded by the use of such devices, and have reported their use has enhanced patient encounters [12]. M-learning devices are perceived to increase opportunities to use clinical experiences as learning opportunities [10] and allow students to make the best use of downtime between clinical activities [9, 16, 17]. The use of mobile devices amongst medical students has also been linked with improved performance in exams [19, 20].

However, studies have identified significant practical and social limitations to the use of such devices – notably poor internet access in clinical areas [10, 12, 17, 21] and a lack of perceived acceptance by patients and clinicians [7, 10, 15, 16, 22–26]. Further barriers include a lack of robust technical support [12], unease about information privacy [1, 15] and concerns that devices may be lost, damaged or stolen [8, 12, 27].

Whilst popular with students, it has been suggested that adopting mLearning may not necessarily have a positive impact on learning outcomes. Patil and colleagues demonstrated that despite students’ positive attitudes towards mLearning, utilisation of learning materials provided on mobile devices was low [28]. The presence of mobile devices has also been reported to lead to increased disruptions during teaching sessions [1, 24] and higher dependence on seniors for decision making [24].

Limitations to making useful conclusions from previous studies include: sample size variation (15–278) [9, 10]; the short duration of most studies (maximum duration 1 year) [17]; the potential influence of bias when using focus groups to assess outcomes [10, 12, 24, 28]; and a focus on personal digital assistants (PDAs) [11, 22] rather than smartphones/tablets. There is also debate in the literature regarding the limitations of evaluating impacts of mLearning devices in the rapidly evolving technological era.

Given the difficulty in extrapolating consistent conclusions from the existing literature, this study, to gather feedback from a large number of students over a longer period of time, was conducted in a large medical school before making decision about the widespread introduction of mLearning as a teaching strategy.

## Methods

### Aims

This study aimed to evaluate the impact of mLearning devices provided to support placement-based learning by gathering feedback from a large group of students, over a long observational period, in a naturalistic setting. The study objectives were:

1. To identify the attitudes of the students, as well as the perceived reaction of surrounding clinicians and patients towards the use of mLearning devices in clinical learning settings
2. To identify students’ perceived impacts of mLearning devices as an adjunct to learning in clinical settings
3. To identify whether mLearning devices have an impact on the reported length or efficiency of students studying hours
4. To identify any significant limitations to the use of devices in healthcare education

Sample, Data collection and Study Period:

Over three academic years (October 2013 – July 2016) 18 cycles of medical students (*n* = 275) undertaking their six week ‘Digestive Health’ placement covering General Surgery and Gastroenterology at a single, large teaching hospital in London were offered a tablet device to use during their placement. Apple iPad Minis (2013 model) were used for each cohort. The device contained various pre-loaded applications (Additional file 1: Appendix S1) chosen by teaching staff, but allowed participants full autonomy to load any further apps of their choice. Devices were allocated and set up at an ‘induction’ session as part of an overall introduction to the placement and students received ongoing support from an ad-hoc peer-led ‘mLearning Support Clinic’, contactable via email.

A Google Form survey questionnaire (Additional file 2: Appendix S2 and Additional file 3: Appendix S3), consisting of a mixture of Likert scale questions assessing perceived advantages and disadvantages, simple Yes/No responses, and free text boxes was designed based on finding in the empirical literature [8, 15, 24, 26] and the outcomes of a student focus group and pilot survey. The questionnaire was provided on the device and completed in the first ‘induction’ session and students were strongly encouraged to complete the follow-up version of the questionnaire (Additional file 2: Appendix S2 and Additional file 3: Appendix S3).

During the observational period, emergent data suggested improved efficiency of work may be a significant unanticipated benefit and therefore from 2014 onwards an additional question was added to the pre- and post-intervention questionnaires exploring this domain. Furthermore, during the study, expanded WiFi access was introduced in the teaching hospital that was the site of the study, with the potential to improve experiences of cohorts taking part in the study.

### Analysis

Given the variation during the data collection period, the analysis period was broken down into ‘Phase I’ (from Oct. ‘13 – Jul. ‘14) and ‘Phase II’ (from Oct. ‘14 – Jul. ‘16). Phase II data was analysed separately as an additional question regarding efficiency of work was added to this cohorts’ questionnaire.

A second subgroup analysis was applied to ‘Pre-expanded WiFi’ (Oct. ‘13 – Jan. ‘15) vs. ‘Post-expanded WiFi’ (Jan. ‘15- Jul. ‘16) because of the infrastructure changes identified above.

The analysis of quantitative questionnaire data was conducted using STATA (v14). Data were assessed for variance and whether the data was parametric or non-parametric. to decide upon appropriateness of statistical test. Continuous variables were assessed using t-tests, categorical and nominal variables were assessed using chi squared or Fisher’s exact where sample size was deemed small. Wilcoxon rank sum tests was used for comparison of two groups where variables were non-parametrically distributed.

The free text data was recorded in a single Microsoft® Excel spreadsheet. Systematic thematic analysis was applied: two authors (DG and TC) independently analysed the data and identified preliminary themes. These were reviewed and clustered by DG, TC and BF and a final set of themes was agreed and then applied to the full data set. [29].

## Results

### Demographics

Over a 33-month period from October 2013 to July 2016, 275 participants (138 male, 137 female), with an average age of 22.2 years old (Range 21–31), undertaking a surgical rotation in a single London teaching hospital were recruited (Table 1). This represented 72.9% of the total students completing the rotation (*n* = 377). 100% (*n* = 275) of participants completed the ‘pre-intervention’ questionnaire and 79% (*n* = 217) completed the ‘post-intervention’ questionnaire.

**Table 1:** Participants completing questionnaire

	Pre-intervention	Post-intervention
Phase I	101	98
Phase II	174	119
Total	***275***	***217***

Significant *p*< 0.05 are in bold italic

### Impact on work hours

The average number of hours spent using the device was reported as approximately two hours per day (range < 1 h to > 12 h). Whilst there was no significant change in the reported hours of private study by participants before and after the placement to the direct question *‘How many hours do you spend on personal study each week*?’ reported at 11.6 h and 12.7 h per week (+ 1.1 *p* = 0.102) pre- and post-intervention respectively, in response to a separate question concerning their overall time spent studying during the week, 61% (*n* = 133) of students felt the tablet device did increase with an average reported increase of 3.1 h/week.

### Utility

Students varied in where and when they used the devices as an adjunct for learning. The most useful time and place identified, on a scale of 1–5 (where 5 is the most useful), was ‘*in the student hub’* (3.8) followed by ‘*in the library’* (3.7), ‘*in spare time between clinical sessions’* (3.6), and *‘at home’* (3.5) compared to the lowest score of ‘*Clerking on the ward*’ (2.2). Rather than as a support for clinical learning, students were mainly using the device in informal and private settings.

Free text (FT) comments suggested some of this may be due to poor internet access in clinical areas;

*‘I don’t think that iPads will be particularly useful for clinical students until there is Internet access throughout the hospital ‘.*

*FT1*

But may also be due to how students use mobile devices for learning generally:

*‘I personally think that it is a great device to do some productive work between clinical sessions and wards, but not during the sessions themselves’.*

*FT2*

### Impact on work efficiency

**Fig. 1:**
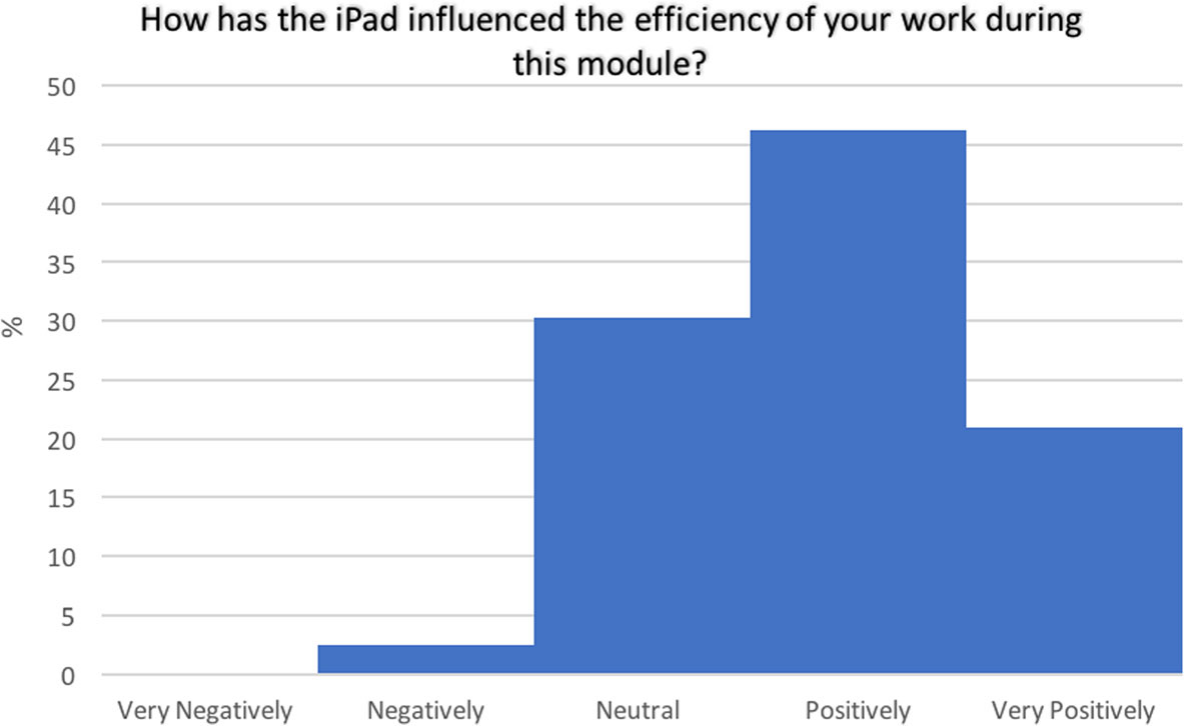
Perceived effect of the iPad on efficiency of work

From 2014 onwards, an additional question concerning efficacy was included. Analysis of the responses from this subgroup revealed 67% (*n* = 80) of students felt that the mLearning device made the hours they spent working more efficient (Fig. 1).

### Internet access

Good internet access was central to the usefulness of the devices. Whilst 80% (*n* = 100) of students reported internet access as a significant limiting factor in the early part of the study prior, once WiFi access was expanded, this figure decreased to 56.5% (*n* = 52) (*p* < 0.001). Despite this expansion, only 10.9% (*n* = 10) of students felt that they could access internet wherever they needed it, with 78.3% (*n* = 72) of students suggesting that the WiFi access required further expansion in the clinical environment to aid mLearning. Alternatively, a small percentage of students voted for the Medical School to provide a SIM or contribute towards data costs (10.9% (*n* = 10) and 0% (*n* = 0) respectively) (Table 2).

**Table 2:** Support of Internet Access

Q: How should the Medical school support internet access			
	Pre-expanded WiFi % (*n* = 125)	Post-expanded WiFi % (*n* = 92)	%Change (*p*)
No need- I can find internet wherever I need it	0.8 (1)	10.9 (10)	10.1 (0.001)
The Medical School should increase WiFi access in the clinical environment	88.0 (110)	78.3 (72)	−9.7 (0.0629)
The Medical School should provide a SIM for tablet devices with a data allowance.	9.6 (12)	10.9 (10)	1.3 (0.822)
The Medical School should contribute towards students’ data costs, via tethering from their smartphone or their own SIM.	1.6 (2)	0.00 (0)	−1.6 (0.509)

Free text feedback also identified WiFi availability as the central issue impacting on the usefulness or otherwise of the devices as an adjunct to learning:

*‘The size of the iPad and availability of some books were really great. But it was mainly the poor Internet connection that lead me to prefer sticking back to books and pen and paper which is what I’m more comfortable with’.*

*FT3*

### Perceived advantages and disadvantages

Participants were asked a series of questions concerning the perceived advantages and disadvantages of the use of a mobile device both before and after the study period. Answers were rated on a 5-point Likert scale from ‘Strongly Disagree’ [1] to ‘Strongly Agree’ [5]. To gauge if the devices elicited a change in attitude, an unpaired t-test was used to compare the average pre- vs. post-intervention response to each question.

#### Advantages

By the end of the study the students agreed with all advantages suggested in the questionnaire (mean score > 3), most notably regarding speed of information access, administration, multimedia learning and up-to-date resources (mean score > 4).

Free text comments also identified the additional advantages of the size and portability of the iPad mini device and the bonus of free access to core texts as e-books on the device.

#### Disadvantages

All disadvantages suggested in the questionnaire showed a significant decrease (*p* < 0.05) in score from pre- to post-intervention, showing perceptions of disadvantages were reduced by device use. Users dismissed 10 out of 13 proposed disadvantages following the period of use (score < 3). The largest decreases included those relating to negative perceptions by patients or relatives and clinicians (− 0.93 and − 0.89 respectively). The smallest attitude changes were found in domains relating to superficial learning (− 0.31), cost of device (− 0.37), information overload (− 0.42) and risk of loss or theft (− 0.43) (Table 3).

**Table 3:** Percieved Advantages and Disadvantages

	Average post-intervention response	Change (p)
Perceived Advantages:		
Producing better notes	3.44	***0.31*** (< 0.01)
Generates more opportunities for group learning	3.63	0.12 (0.12)
Ability to link different sources of information	3.93	− 0.08 (0.28)
More efficient use of study time	3.97	0.14 (0.06)
Access to more up-to-date resources	4.09	***−0.18*** (< 0.01)
Access to multimedia learning	4.17	***−0.15*** (< 0.05)
Ease of everyday administrative tasks	4.25	−***0.18*** (< 0.05)
Easier and faster to find information	4.28	***−0.25*** (< 0.001)
Perceived Disadvantages:		
Encourages acquisition of superficial layers of knowledge rather than in-depth learning	2.41	***−0.31*** (< 0.001)
Cost of device	3.65	***−0.37*** (< 0.001)
Information overload	2.47	***−0.42*** (< 0.001)
Risk of loss or theft	3.49	***−0.43*** (< 0.001)
Need to account for new professional/personal behaviours	2.71	***−0.46*** (< 0.001)
Information not always accessible due to absence of internet connection	3.69	***−0.48*** (< 0.001)
Difficult to ascertain quality and accuracy of available apps	2.85	***−0.52*** (< 0.001)
Risk of unauthorised access to personal data	2.83	***−0.55*** (< 0.001)
Distracts from communicating with patients	2.41	***−0.55*** (< 0.001)
Distracts from the clinical environment	2.50	***−0.62*** (< 0.001)
Reliance on mobile device rather than own initiative/skills	2.56	−***0.64*** (< 0.001)
Negative perceptions by clinicians	2.52	***−0.89*** (< 0.001)
Negative perception by patients or their relatives	2.64	***−0.93*** (< 0.001)

Significant *p*< 0.05 are in bold italic

Some students also felt the devices were overloaded or slow:

‘There were too many apps on the device, and not many of them were used. They also took up memory and so made the device much slower….moreover, most students use Wikipedia as a first point of reference for many things. This just requires a browser rather than an app’.

*FT4*

### Device provision

Students were asked whether they thought the medical school should provide a device or support the use of their own device (Table 4), with opinion changing over the study period. This opinion change corresponded to an increase in students already owning mLearning devices. By phase II of the study 92.5% (*n* = 161) of students reported they had an alternative device they could use for mLearning, a significant 13.4% increase (*p* = 0.0036) from phase I.

**Table 4:** How should the Medical school support the use of tablet devices in medical education?

	Phase I % (*n*)	Phase II % (*n*)	% Change (*p*)
Provide a device	57.1 (56)	45.4 (54)	−11.8 (0.085)
Provide apps and support the use of own device	37.8 (36)	36.7 (46)	−1.9 (0.772)
Let students decide whether to use a tablet device	6.1 (6)	16.0 (19)	9.8 (0.019)

Free text feedback from students showed a preference for ‘bring your own device’:

*‘I rarely used my device because I found my Android tablet faster and easier to use. I liked the idea of the apps and information on the UCL tablet but in practice, rarely used it. I would prefer support for my own device.’*

*FT5*

*‘I didn’t use the iPad at all because it offers nothing that a smartphone doesn’t offer, whilst being too large to fit in a pocket’.*

*FT6*

### Troubleshooting

Over the entire study, 44 of the students reported technical issues. Of these, 23 were resolved in less than 10 min without any outside help, and only four issues could not be resolved with help.

## Discussion

The positive impact of the devices on the students’ perceived efficiency of work, consistent with Wallace and colleagues’ finding of efficient use of time with mobile computing devices [15], suggests students made good use of the mobile devices. They did so in a variety of settings: clinical spaces; libraries and social spaces; but predominantly in areas with good WiFi access and away from patient encounters, consistent with existing studies finding most use between scheduled activities [16, 17, 19, 22] due to the flexibility and portability of the devices [1, 12, 27]. The inconsistency with the reported change in time spent on ‘personal study’ each week (no significant increase) when compared to 61% of students reporting the device increasing overall time spent studying may be due to a perceived difference between ‘personal study’ and total time studying with the extra time spent using the devices between clinical commitments not included in ‘personal study’.

WiFi expectation far exceeded provision. Faster access to the internet is seen as a major benefit of mLearning devices [15] and even after increased connectivity, our results showed students still felt limited by internet access, which is similar to findings by other authors [12, 21, 27]. These results suggest that internet connectivity in clinical spaces will play a major role in restricting the potential of such devices for placement-based education and is mentioned by Deutsch and colleagues (2016) as a key factor in planning before implementation of a mLearning program [30].

Students were already aware of the potential positive impact of mLearning devices in the clinical setting, and these existing views were reinforced over the study period. Initial concerns about possible disadvantages of devices in clinical settings were largely unfounded, notably the perceived reaction by clinicians, patients or their relatives. Previous studies have also reported expected negative reactions to mLearning devices from this population [12, 16, 17, 26]. However, it appears that if the devices are explained/clearly for professional use then patients are amenable to their use [12, 23]. The supply of university branded cases to each student in this study may have assisted in reducing negative reactions. Interestingly, despite these students, like those in the study by Alegría and colleagues [9], remaining concerned about loss, damage or theft, over the entire study this was limited to one damaged screen and seven lost charging cables.

There was no clear agreement from the students as to whether they should be provided with a device or supported to use of their own device. The significant reported change in existing ownership of a mLearning device over this relatively short period highlights the rapidly evolving landscape of technology. However, the authors agree with findings by Friedericks and colleagues that the increased size of the tablet screen may provide additional functionality compared to a smartphone [31] and universal and equitable access to a tablet device may only be achieved via University provision.

Our study also demonstrated that the technological life-span of the devices was sufficient for the full three years of the study (equivalent to an entire clinical cycle of a medical student) with no instances of device obsolescence. It was beyond the scope of this study to examine whether a single device would be suitable for an entire degree (5–6 years), but the rate of advance of consumer electronic products suggests this is unlikely.

Although there were concerns by the project team that troubleshooting assistance may be labour intensive, the cohort using the devices required minimal outside support. There are suggestions that peer-peer technical support is prevalent amongst this population [21, 25] and this support network may explain the low reliance on a formal support network.

### Limitations

Given the setting, the research question and the naturalistic methodological approach the main limitation was the lack of control group. The anonymous nature of the questionnaire made it impossible to identify and follow up the 58 survey non-responders. The short intervention period of six weeks may not have allowed students to capitalise on the device’s potential. The generalisability is also limited by performing the study at a single site, on a single clinical rotation using one brand of device. Finally, there is a danger of recall bias as students were asked to self-report their usage, rather than usage telemetry being directly recorded from the devices and our study may have shown selection bias of tech-savvy students.

## Conclusion

Medical students embrace mLearning devices in the clinical setting and whilst it remains unclear if the total length of time spent on study increases, the devices had a positive effect on the perceived efficiency of students’ work. Importantly, contrary to much of the literature, students disagreed that patients or clinicians reacted negatively to device use in the clinical setting (Table 3). However, as is commonly reported elsewhere, WiFi availability, particularly in clinical areas, proved essential but limited, which needs to be addressed by medical schools in conjunction with placement providers. It remains unclear from this study if students prefer to be given a pre-loaded device for their clinical training or to have their own device supported by access to educational resources. However, it was shown the devices provided were robust enough to support the typical 3-years of placement based education.

Given societal changes in the use of smart devices, the authors agree with others’ suggestion that mLearning will become a ubiquitous component of the undergraduate medical students learning [15]. Medical schools should make purposeful plans to incorporate mLearning, while being mindful of how students use the devices and the fundamental need for universal access to the internet to ensure useful learning can take place.

## Additional Files

Additional File 1: Appendix S1. List of applications pre-loaded on iPad devices. (DOCX 99 kb)

Additional File 2: Appendix S2. Selected survey questions with data analysed within this manuscript. (DOCX 63 kb)

Additional File 3: Appendix S3. Full list of questions in post-survey questionnaire. (DOCX 95 kb)

## Abbreviations

eBook: Electronic Book
FT: Free Text Comment
mLearning: Mobile Learning
PDAs: Personal Digital Assistants
