# Supplementary material for: Mobile learning in medicine: an evaluation of attitudes and behaviours of medical students
Source: BMC Med Educ. 2018 Jun 27;18:152. doi: 10.1186/s12909-018-1264-5 (PMC6020287; doi:10.1186/s12909-018-1264-5)
Supplement: Supplementary file 1 — Appendix S1. List of applications pre-loaded on iPad devices. (DOCX 99 kb) [file 12909_2018_1264_MOESM1_ESM.docx]

**Appendix 1: List of applications pre-loaded on iPad devices**

**Books**

**Dr. Companion Mobile Medical** available at: [*https://itunes.apple.com/us/app/medhand-mobile-libraries/id557073873?mt=8*](https://itunes.apple.com/us/app/medhand-mobile-libraries/id557073873?mt=8)

**The Blood Gas Handbook** available at:

<https://itunes.apple.com/us/app/avoid-preanalytical-errors/id537041030?mt=8>

**Guidance**

**NICE BNF** available at:

<https://itunes.apple.com/gb/app/bnf-publications/id1045514038?mt=8>

**NICE BNFC** available at:

<https://itunes.apple.com/gb/app/bnf-publications/id1045514038?mt=8>

**almostadoctor** information available at:

[https://www.imedicalapps.com/2012/03/almostadoctor-app-brings-free-medical-encyclopaedia-iphone/#](https://www.imedicalapps.com/2012/03/almostadoctor-app-brings-free-medical-encyclopaedia-iphone/)

**BMJ Best Practice** available at:

<https://itunes.apple.com/gb/app/bmj-best-practice/id1105379489?mt=8>

**Medscape** available at:

<https://www.medscape.com/public/medscapeapp>

**SIGN Guidelines** available at:

<http://www.sign.ac.uk/sign-apps.html>

**Reference**

**UCL Go!** Available at:

<http://www.ucl.ac.uk/isd/services/websites-apps/apps/ucl-go>

**UCLiMap** available at:

<https://itunes.apple.com/WebObjects/MZStore.woa/wa/viewArtist?id=315139508>

**Wikipedia Mobile** available at:

<https://itunes.apple.com/us/app/wikipedia/id324715238?mt=8>

**Youtube** available at:

<https://itunes.apple.com/gb/app/youtube-watch-listen-stream/id544007664?mt=8>

**iBooks** available at:

<https://itunes.apple.com/ke/app/ibooks/id364709193?mt=8>

**iTunes U** available at:

<https://itunes.apple.com/gb/app/itunes-u/id490217893?mt=8>

**Eponyms (for students)** available at:

<https://itunes.apple.com/us/app/eponyms-for-students/id286025430?mt=8>

**Scripts and Notes**

**Calculate (Medical Calculator)** available at:

<https://itunes.apple.com/us/app/calculate-by-qxmd/id361811483?mt=8>

**CliniCalc Medical Calculator** available at:

<https://itunes.apple.com/gb/app/clinicalc-medical-calculator/id353404314?mt=8>

**MyScript Calculator** available at:

<https://itunes.apple.com/us/app/myscript-calculator/id1304488725?mt=8>

**Evernote** available at:

<https://itunes.apple.com/app/evernote/id281796108?mt=8>

**Penultimate** available at:

<https://evernote.com/products/penultimate>

**Logistics**

**Adobe Reader** available at:

<https://itunes.apple.com/gb/app/adobe-acrobat-reader/id469337564?mt=8>

**Bluefire Reader** available at:

<https://itunes.apple.com/us/app/bluefire-reader/id394275498?mt=8>

**Dropbox** available at:

<https://www.dropbox.com/ipad>

**Find My iPhone** available at:

<https://itunes.apple.com/gb/app/find-my-iphone/id376101648?mt=8>

**Cisco AnyConnect** available at:

<https://itunes.apple.com/us/app/cisco-anyconnect/id1135064690?mt=8>

**PDF Master** available at:

<https://itunes.apple.com/us/app/pdf-master-annotate-pdfs-sign-documents-fill-forms/id884813085?mt=8>
