# Supplementary material for: Mobile learning in medicine: an evaluation of attitudes and behaviours of medical students
Source: BMC Med Educ. 2018 Jun 27;18:152. doi: 10.1186/s12909-018-1264-5 (PMC6020287; doi:10.1186/s12909-018-1264-5)
Supplement: Supplementary file 2 — Appendix S2. Selected survey questions with data analysed within this manuscript. (DOCX 63 kb) [file 12909_2018_1264_MOESM2_ESM.docx]

**Appendix 2: Survey questions (with data analysed within the manuscript)**

These have been grouped under lettered headings according to their corresponding use in the analysis section of the paper. Possible answer options available for students are given here in bullet points.

1. Demographics

**How old are you?**

- X years old

**What is your gender?**

- Male
- Female

1. Work Hours

**How many hours do you spend on personal study each week?**

- - X hours

**Do you think your UCL tablet device increased the overall time you spent studying?**

- - No - I spent less time studying
  - No - I spent the same amount of time studying
  - Yes – X hours of additional studying per week

1. Utility

**How useful did you find your UCL tablet device in the following settings…?**

Here each bullet point option was asked as a separate question with possible responses on a 5-point Likert scale:

1-Not useful, 2-Rarely useful, 3-Useful, 4-Quite useful, 5-Very useful

- Teaching on the wards
- Clerking on the ward
- Outpatient Clinic
- In Lectures
- In Tutorials
- In the library
- In the Student Hub
- In spare time between clinical sessions
- On your commute
- At home

1. Work efficiency

**How has the iPad influenced the efficiency of your work during this module?**

- - Very negatively
  - Negatively
  - Neutral
  - Positively
  - Very positively

1. Internet access

**Did a lack of internet connectivity prevent you from using your tablet as intended?**

- - Yes
  - No

**How should the Medical school support internet access**

- - No need- I can find internet wherever I need it
  - The Medical School should increase WiFi access in the clinical environment
  - The Medical School should provide a SIM for tablet devices with a data allowance.
  - The Medical School should contribute towards students' data costs, via tethering from their smartphone or their own SIM

1. Perceived advantages vs disadvantages

Here each bullet point option was asked as a separate question with possible responses on a 5-point Likert scale:

1-strongly disagree, 2-disagree, 3-neutral, 4-agree, 5-strongly agree

**What do you think are the main advantages of a mobile device for your medical education?**

- More efficient use of study time
- Generates more opportunities for group learning
- Ability to link different sources of information
- Easier and faster to find information
- Producing better notes
- Access to multimedia learning
- Access to more up-to-date resources
- Ease of everyday administrative tasks

**What do you think are the main disadvantages of a mobile device for your medical education?**

- - Distracts from the clinical environment
  - Encourages acquisition of superficial layers of knowledge rather than in-depth learning
  - Distracts from communicating with patients
  - Information overload
  - Reliance on mobile device rather than own initiative/skills
  - Negative perceptions by clinicians
  - Negative perception by patients or their relatives
  - Risk of unauthorised access to personal data
  - Difficult to ascertain quality and accuracy of available apps
  - Need to account for new professional/personal behaviours
  - Information not always accessible due to absence of internet connection
  - Cost of device
  - Risk of loss or theft

1. Device provision

**Do you already own a device that could be used for mLearning?**

- Yes
- No

**How should the Medical school support the use of tablet devices in medical education?**

- - The Medical School should provide a device.
  - The Medical School should let students make the decision whether to use a tablet device
  - The Medical School should provide apps and support for my own device.

**Are you considering buying a new tablet or smartphone as a result of your experience?**

- Yes
- No

**For those considering buying a new tablet or smartphone… Are you considering purchasing a smartphone or a tablet?**

- Smartphone
- Tablet

1. Free text boxes:

**Please tell us about any specific areas with no WiFi coverage that you would like to see addressed**

**Do you have any more feedback about the make and model of the mLearning device you were issued?**

**Please tell us about any advantages or disadvantages of mobile devices you consider important that are not listed above.**

**Do you have any other thoughts on how a tablet computer might improve student and clinician experience with the ePortfolio?**

**Do you have any final thoughts on the study organisation, or devices, which you would like to share with us?**
