# Supplementary material for: Mobile learning in medicine: an evaluation of attitudes and behaviours of medical students
Source: BMC Med Educ. 2018 Jun 27;18:152. doi: 10.1186/s12909-018-1264-5 (PMC6020287; doi:10.1186/s12909-018-1264-5)
Supplement: Supplementary file 3 — Appendix S3. Full list of questions in post-survey questionnaire. (DOCX 95 kb) [file 12909_2018_1264_MOESM3_ESM.docx]

**Appendix 3: Full list of questions in post-survey questionnaire:**

(List of possible answers removed for simplicity. Data analysis of many questions not included in this paper as was beyond the scope of one manuscript)

1. What is your gender?
2. How old are you?
3. How many hours do you spend on personal study each week?
4. Please tell us about any computing devices you own.
5. Which resources do you use to help you study?
6. Which UCL mLearning device were you loaned?
7. Was the storage capacity on the device sufficient for your educational needs?
8. Did you have any technical problems with your device?
9. Do you have any comments regarding the pre-study induction?
10. Did you use the mLearning Support Clinic?
11. Did screen size limit what you were able to do with your device – portability, reading/watching content, making notes?
12. Do you have any more feedback about the make and model of the UCL mLearning device your were issued?
13. To what extent do you agree with the following statements? [I was worried my device would get lost or damaged during daily use.]
14. To what extent do you agree with the following statements? [I received negative comments from clinicians, patients, colleagues or staff.]
15. To what extent do you agree with the following statements? [I found the device could be a distraction from studying, clerking or communicating with clinicians.]
16. Regarding the mLearning support clinic, to what extent do you agree with the following statements? [The mLearning tutors were knowledgeable on the software and hardware provided.]
17. Regarding the mLearning support clinic, to what extent do you agree with the following statements? [The mLearning tutors were able to solve my technical problems.]
18. Regarding the mLearning support clinic, to what extent do you agree with the following statements? [The mLearning tutors were able to teach me new ways to use my device.]
19. Regarding the mLearning support clinic, to what extent do you agree with the following statements? [I received a timely response to my queries.]
20. Do you have any other comments regarding the mLearning support clinic?
21. How many hours did you spend using your UCL tablet device each day?
22. How much did you use your UCL tablet device in the following settings every week: [Teaching on the wards]
23. How much did you use your UCL tablet device in the following settings every week: [Clerking on the wards]
24. How much did you use your UCL tablet device in the following settings every week: [In outpatient clinics]
25. How much did you use your UCL tablet device in the following settings every week: [In Lectures]
26. How much did you use your UCL tablet device in the following settings every week: [In Tutorials]
27. How much did you use your UCL tablet device in the following settings every week: [In the Library]
28. How much did you use your UCL tablet device in the following settings every week: [In the Student Hub]
29. How much did you use your UCL tablet device in the following settings every week: [In spare time between clinical sessions]
30. How much did you use your UCL tablet device in the following settings every week: [On your commute]
31. How much did you use your UCL tablet device in the following settings every week: [At home]
32. How useful did you find your UCL tablet device in the following settings: [Teaching on the wards]
33. How useful did you find your UCL tablet device in the following settings: [Clerking on the wards]
34. How useful did you find your UCL tablet device in the following settings: [In outpatient clinics]
35. How useful did you find your UCL tablet device in the following settings: [In Lectures]
36. How useful did you find your UCL tablet device in the following settings: [In Tutorials]
37. How useful did you find your UCL tablet device in the following settings: [In the Library]
38. How useful did you find your UCL tablet device in the following settings: [In the Student Hub]
39. How useful did you find your UCL tablet device in the following settings: [In spare time between clinical sessions]
40. How useful did you find your UCL tablet device in the following settings: [On your commute]
41. How useful did you find your UCL tablet device in the following settings: [At home]
42. How many features/apps did you use on your UCL tablet device each day?
43. Do you think your UCL tablet device increased the overall time you spent studying?
44. For consuming content (eg reading textbooks, watching videos), how much did you use your UCL tablet device relative to traditional resources such as textbooks or laptops/desktops?
45. For making content (eg notes, presentations), how much did you use your UCL tablet device relative to traditional resources such as a pen and paper or laptops/desktops?
46. Did a lack of internet connectivity prevent you from using your UCL tablet as you intended?
47. Please tell us about the availability of internet access in the following settings. [By the bedside.]
48. Please tell us about the availability of internet access in the following settings. [By the nurse's station.]
49. Please tell us about the availability of internet access in the following settings. [In the doctor's office.]
50. Please tell us about the availability of internet access in the following settings. [In seminar rooms.]
51. Please tell us about the availability of internet access in the following settings. [In clinic.]
52. Please tell us about the availability of internet access in the following settings. [In operating theatres.]
53. Please tell us about the availability of internet access in the following settings. [In lecture theatres.]
54. Please tell us about the availability of internet access in the following settings. [In corridors.]
55. Please tell us about the availability of internet access in the following settings. [In GP surgeries and other off-site activities.]
56. Please tell us about any specific areas with no wifi coverage that you would like to see addressed.
57. On site, did you supplement wifi internet access with your own internet access in areas where Eduroam wasn't available?
58. Did you incur any additional costs by supplementing internet access to your UCL tablet device?
59. Which resources did you use more as a result of your UCL tablet device? [Textbooks]
60. Which resources did you use more as a result of your UCL tablet device? [Lecture Slides]
61. Which resources did you use more as a result of your UCL tablet device? [eBooks]
62. Which resources did you use more as a result of your UCL tablet device? [Note taking (pen & paper or electronic)]
63. Which resources did you use more as a result of your UCL tablet device? [Websites]
64. Which resources did you use more as a result of your UCL tablet device? [Search engines]
65. Which resources did you use more as a result of your UCL tablet device? [National Guidelines (NICE, BTS, etc...)]
66. Which resources did you use more as a result of your UCL tablet device? [Online question banks]
67. Which resources did you use more as a result of your UCL tablet device? [Journal articles]
68. Which resources did you use more as a result of your UCL tablet device? [Clinical Calculators]
69. Which resources did you use more as a result of your UCL tablet device? [Podcasts]
70. Which resources did you use more as a result of your UCL tablet device? [Video tutorials]
71. Which resources did you use more as a result of your UCL tablet device? [Forums]
72. Which resources did you use more as a result of your UCL tablet device? [Mind-mapping]
73. Which resources did you use more as a result of your UCL tablet device? [Reading lists]
74. Please rate the impact of your UCL tablet in the following areas: [Time management]
75. Please rate the impact of your UCL tablet in the following areas: [Organisation]
76. Please rate the impact of your UCL tablet in the following areas: [Communication with medical student peers]
77. Please rate the impact of your UCL tablet in the following areas: [Communication with clinicians]
78. Please rate the impact of your UCL tablet in the following areas: [ePortfolio access]
79. Please rate the impact of your UCL tablet in the following areas: [Use of Library Resources (eBooks, Module Reading List)]
80. Do you already own a device that could be used for mLearning?
81. Are you making more use of your existing mobile device as a result of your UCL tablet device?
82. Since starting the mLearning pilot, are there any new resources you've used on your own smartphone or tablet?
83. Tell us about any interactive training tools you used, either on your UCL tablet or your own device.
84. Tell us about any medical reference tools you used, either on your UCL tablet or your own device.
85. Tell us about any medical textbook apps you used, either on your UCL tablet or your own device.
86. Tell us about any question banks you used, either on your UCL tablet or your own device.
87. Tell us about any other productivity apps you used.
88. Tell us about any leisure apps you used:
89. Please tell us about any useful functions, apps or links not listed above.
90. Please tell use about any useful functions, apps or links that you think should be preloaded on the devices.
91. Tell us about the usefulness of each of the preloaded apps or links: [NICE CKS]
92. Tell us about the usefulness of each of the preloaded apps or links: [BMJ Best Practice]
93. Tell us about the usefulness of each of the preloaded apps or links: [BMJ Differentials]
94. Tell us about the usefulness of each of the preloaded apps or links: [UCL Go!]
95. Tell us about the usefulness of each of the preloaded apps or links: [Bluefire Reader]
96. Tell us about the usefulness of each of the preloaded apps or links: [SIGN Guidelines]
97. Tell us about the usefulness of each of the preloaded apps or links: [NICE Guidelines]
98. Tell us about the usefulness of each of the preloaded apps or links: [BNF]
99. Tell us about the usefulness of each of the preloaded apps or links: [Children's BNF]
100. Tell us about the usefulness of each of the preloaded apps or links: [Clinicalc]
101. Tell us about the usefulness of each of the preloaded apps or links: [Blood Gas]
102. Tell us about the usefulness of each of the preloaded apps or links: [iTunes U]
103. Tell us about the usefulness of each of the preloaded apps or links: [UCL iMap]
104. Tell us about the usefulness of each of the preloaded apps or links: [Dropbox]
105. Tell us about the usefulness of each of the preloaded apps or links: [Adobe Reader]
106. Tell us about the usefulness of each of the preloaded apps or links: [Evernote]
107. Tell us about the usefulness of each of the preloaded apps or links: [Calculator]
108. Tell us about the usefulness of each of the preloaded apps or links: [AnyConnect]
109. Tell us about the usefulness of each of the preloaded apps or links: [Penultimate]
110. Tell us about the usefulness of each of the preloaded apps or links: [Qx Calculate]
111. Tell us about the usefulness of each of the preloaded apps or links: [PDF Master]
112. Tell us about the usefulness of each of the preloaded apps or links: [UCL Reading List Link]
113. Tell us about the usefulness of each of the preloaded apps or links: [UCL Moodle Link]
114. Tell us about the usefulness of each of the preloaded apps or links: [ePortfolio Link]
115. Tell us about the usefulness of each of the preloaded apps or links: [Dawsonera eBooks link]
116. Tell us about the usefulness of each of the preloaded apps or links: [Webpath Link]
117. Tell us about the usefulness of each of the preloaded apps or links: [Radiology Masterclass Link]
118. Tell us about the usefulness of each of the preloaded DrCompanion Medhand Textbooks: [Oxford Handbook of Clinical Medicine]
119. Tell us about the usefulness of each of the preloaded DrCompanion Medhand Textbooks: [Oxford Handbook of Clinical Specialties]
120. Tell us about the usefulness of each of the preloaded DrCompanion Medhand Textbooks: [Oxford Handbook of Clinical Examination and Practical Skills.]
121. Tell us about the usefulness of each of the preloaded DrCompanion Medhand Textbooks: [Oxford Handbook of Clinical Surgery]
122. Did you use the Dawsonera online eBooks?
123. Tell us any other ways in which you accessed textbooks on your UCL Tablet.
124. To what extent do you agree with the following statements? [It was easy to find the eBook I was looking for.]
125. To what extent do you agree with the following statements? [The book was easy to download to my tablet device.]
126. To what extent do you agree with the following statements? [The book was easily accessible on my device once downloaded]
127. Do you have any other feedback on the Dawsonera online eBooks?
128. Please tell us about any titles you would like to see added to UCL Library's eBooks collection.
129. What do you think are the main advantages of a mobile device for your medical education? [More efficient use of study time.]
130. What do you think are the main advantages of a mobile device for your medical education? [Easier and faster to find information.]
131. What do you think are the main advantages of a mobile device for your medical education? [Producing better notes.]
132. What do you think are the main advantages of a mobile device for your medical education? [eBooks less expensive than textbooks.]
133. What do you think are the main advantages of a mobile device for your medical education? [Easier to carry than books.]
134. What do you think are the main advantages of a mobile device for your medical education? [Greater opportunities for group learning.]
135. What do you think are the main advantages of a mobile device for your medical education? [Access to multimedia learning resources.]
136. What do you think are the main advantages of a mobile device for your medical education? [Access to more up-to-date resources.]
137. What do you think are the main advantages of a mobile device for your medical education? [Ability to link different sources of information.]
138. What do you think are the main advantages of a mobile device for your medical education? [Ease of everyday administrative tasks (email, calendar, notes, etc...).]
139. What do you think are the main disadvantages of a mobile device for your medical education? [Distracts from clinical environment.]
140. What do you think are the main disadvantages of a mobile device for your medical education? [Distracts from communicating with patients.]
141. What do you think are the main disadvantages of a mobile device for your medical education? [Encourages acquisition of superficial layers of knowledge rather than in depth learning.]
142. What do you think are the main disadvantages of a mobile device for your medical education? [Information overload.]
143. What do you think are the main disadvantages of a mobile device for your medical education? [Reliance on mobile device rather than own initiative/skills.]
144. What do you think are the main disadvantages of a mobile device for your medical education? [Negative perception by clinicians.]
145. What do you think are the main disadvantages of a mobile device for your medical education? [Negative perception by patients or their relatives.]
146. What do you think are the main disadvantages of a mobile device for your medical education? [Information not always accessible due to absence of internet connection.]
147. What do you think are the main disadvantages of a mobile device for your medical education? [Cost of device.]
148. What do you think are the main disadvantages of a mobile device for your medical education? [Risk of damage, loss or theft.]
149. What do you think are the main disadvantages of a mobile device for your medical education? [Risk of unauthorised access to personal data.]
150. What do you think are the main disadvantages of a mobile device for your medical education? [Difficult to ascertain the quality and accuracy of available Apps.]
151. What do you think are the main disadvantages of a mobile device for your medical education? [Need to account for new professional and personal behaviours.]
152. Please tell us about any advantages or disadvantages of mobile devices you consider important that are not listed above.
153. Are you considering buying a new tablet or smartphone as a result of your experience?
154. Are you considering purchasing a smartphone or a tablet?
155. Which features of your UCL tablet device have made you consider the purchase?
156. Which device are you considering purchasing?
157. How have you accessed your ePortfolio?
158. How have you completed ePortfolio assessments so far?
159. If appropriate, have you asked clinicians to complete ePortfolio assessments on your tablet device?
160. In your experience, why have assessors not been able to complete assessments on your tablet device?
161. What are the advantages of completing ePortfolio assessments with the clinician present instead of sending an email ticket? [I receive more detailed feedback.]
162. What are the advantages of completing ePortfolio assessments with the clinician present instead of sending an email ticket? [There is less chance the clinician will not fill out the assessment.]
163. What are the disadvantages of completing ePortfolio assessments with the clinician present instead of sending an email ticket? [Completing the assessment with the clinician is more time consuming than an email ticket.]
164. What are the disadvantages of completing ePortfolio assessments with the clinician present instead of sending an email ticket? [I'm more likely to receive feedback in the company of my peers.]
165. Do you have any other thoughts on how a tablet computer might improve student and clinician experience with the ePortfolio?
166. Please rate the usefulness of the following UCL Library resources: [UCL Library reading list]
167. Please rate the usefulness of the following UCL Library resources: [UCL Library eBooks]
168. Please tell us how you think either the reading list or the eBooks collection could be improved. How has the iPad influenced the efficiency of your work during this module?
169. How should UCL Medical School support the use of tablet devices in medical education?
170. Which kind of mobile learning device should the Medical School provide or support?
171. How should the Medical School support internet access from your tablet device?
172. Which year group do you think would benefit most from having a mobile device provided for their medical education?
173. Thank you for taking the time to complete this survey! Do you have any final thoughts on the pilot study organisation, or devices, which you would like to share with us?
